# 12/15-Lipoxygenase Deficiency Impairs Neutrophil Granulopoiesis and Lung Proinflammatory Responses to *Aspergillus fumigatus*

**DOI:** 10.4049/jimmunol.1900808

**Published:** 2020-02-26

**Authors:** Joseph J. Mackel, Jaleesa M. Garth, Jonathan P. Blackburn, MaryJane Jones, Chad Steele

**Affiliations:** * Department of Medicine, University of Alabama at Birmingham, Birmingham, AL 35294;; † Department of Microbiology and Immunology, School of Medicine, Tulane University, New Orleans, LA 70112

## Abstract

Development of invasive aspergillosis correlates with impairments in innate immunity. We and others have recently shown that arachidonic acid metabolism pathways, specifically the cyclooxygenase-2 (COX-2) and 5-lipoxygenase (5-LOX) pathways, participate in the induction of protective innate immune responses during invasive aspergillosis. Based on the high degree of cooperation and interconnection within the eicosanoid network, we hypothesized that 12/15-LOX is also active during invasive aspergillosis. We report in this study that mice deficient in the gene encoding 12/15-LOX (*Alox15*) are profoundly susceptible to invasive aspergillosis. Decreased survival correlated with increased fungal burden and evidence of increased lung damage. These defects were associated with very early (6 and 12 h) 12/15-LOX-dependent inflammatory cytokine (IL-1α, IL-1β, and TNF-α) and chemokine (CCL3 and CCL4) production. Neutrophil levels in the lung were blunted in the absence of 12/15-LOX, although neutrophil antifungal activity was intact. However, lower neutrophil levels in the lungs of *Alox15*^−/−^ mice were not a result of impaired recruitment or survival; rather, *Alox15*^−/−^ mice demonstrated impaired neutrophil granulopoiesis in the bone marrow intrinsically and after fungal exposure. Employing a lower inoculum to allow for better survival allowed the identification of 12/15-LOX-dependent induction of IL-17A and IL-22. Impaired IL-17A and IL-22 production correlated with reduced invariant NKT cell numbers as well as lower IL-23 levels. Together, these data indicate that 12/15-LOX is a critical player in induction of the earliest aspects of the innate immune response to *Aspergillus fumigatus*.

Conidia of the opportunistic fungal pathogen *Aspergillus fumigatus* are sufficiently small and buoyant to reach the lower respiratory tract and have the potential to germinate into large, tissue-destructive hyphae. Immunodeficiencies leading to failure of the innate immune processes that normally control this growth may result in several conditions, the most severe being invasive aspergillosis (IA). In IA, uncontrolled *Aspergillus* growth results in tissue damage and potential dissemination to other organs. Some progress has been made in reducing fatality rates in groups at increased risk for IA, including solid organ transplant recipients and hematological malignancy patients, although current rates still exceed 20% for both groups (1, 2). Current prophylactic antifungal protocols have failed to effectively prevent infections in these groups (3, 4). The ability to target or activate necessary host defense pathways without disrupting the primary treatment modality may improve outcomes. In addition to traditional risk concerns, a newer concern is that climate change may increase the risk of infection by fungal pathogens because of increased selection for thermotolerant species, more exposure during extreme weather events, and increased fungal growth in the environment overall (5, 6).

The eicosanoid class of bioactive lipids are major players in numerous mammalian systems, including the immune system (7). The immunological functions of eicosanoid pathways are of interest, as players in the innate response in the lung because of their rapid action and ability to both drive and resolve inflammation. Distinct eicosanoid species number in the hundreds but belong to a relatively small number of major pathways anchored by key upstream enzymes including cyclooxygenase (COX)-1 and COX-2 and 5-lipoxygenase (LOX) and 12/15-LOX (8). Regarding IA, recent research in our laboratory identified COX-2 as a driver of multiple critical cytokines including IL-17A and IL-22 during IA (9). Another recent report recognized the crucial role of 5-LOX in neutrophil and eosinophil recruitment during IA (10). Interaction between various COX and LOX pathways is a distinguishing feature of eicosanoid biology (8). Thus, the discovery of important roles of COX-2 and 5-LOX during IA begs the question of the function of closely related enzymes, including 12/15-LOX.

12/15-LOX has the biosynthetic capacity to produce multiple families of eicosanoids including proinflammatory hydroxyeicosatetraenoic acids (HETEs) and hepoxilins as well as anti-inflammatory or proresolution resolvins, protectins, and, in cooperation with 5-LOX, lipoxins (11). Investigations of 12/15-LOX during lung infection have primarily associated proresolution products of 12/15-LOX with improved outcomes and proinflammatory products with negative outcomes. Increased levels of the lipoxin A_4_ receptor Alx/Fpr2 were associated with improved clearance of the fungal pathogen *Cryptococcus neoformans* (12). Administration of Resolvin E1 increased bacterial clearance and survival in a model of aspiration pneumonia (13). Conversely, during *Streptococcus* infection 12/15-LOX and its proinflammatory product hepoxilin A_3_ contributed to bacteremia and host death via promotion of neutrophil migration and an associated increase in epithelial permeability (14). In the current report, we describe a role for 12/15-LOX in promotion of protective inflammatory responses, including neutrophil granulopoiesis and cytokine production, which are required for survival and fungal clearance during experimental IA.

## Materials and Methods

### Mice

Male and female, age-matched C57BL/6 mice, 6–8 wk of age, were obtained from The Jackson Laboratory (Bangor, ME). Male and female, age-matched *Alox15*^−/−^ mice were obtained from The Jackson Laboratory (B6.129S2-*Alox15*^*tm1Fun*^/J, stock no. 002778) and bred at Tulane University. All animals were housed in a specific pathogen-free, Association for Assessment and Accreditation of Laboratory Animal Care-certified facility and handled according to Public Health Service Office of Laboratory Animal Welfare policies after review by the Tulane Institutional Animal Care and Use Committee.

### Preparation of *A. fumigatus*: in vivo challenge and lung fungal burden assessment

*A. fumigatus* isolate 13073 (American Type Culture Collection, Manassas, VA) was maintained on potato dextrose agar for 5–7 d at 37°C. Conidia were harvested by washing the culture flask with 50 ml of sterile PBS supplemented with 0.1% Tween 20. The conidia were then passed through a sterile 40-μm nylon membrane to remove hyphal fragments and enumerated on a hemacytometer. For challenge, mice were lightly anesthetized with isoflurane and administered 7 × 10^7^ *A. fumigatus* conidia in a volume of 50 μl intratracheally as previously described (15, 16). Briefly, mice are held in a vertical, upright position, and the tongue is withdrawn from the mouth using forceps. A pipette is used to deliver the 50 μl inoculum to the caudal oropharynx, where normal breathing results in fluid aspiration into the lungs (17). For lung fungal burden analysis, the left lungs were collected at 48 h postexposure and homogenized in 1 ml of PBS. Total RNA was extracted from 0.1 ml of unclarified lung homogenate using the MasterPure Yeast RNA Purification Kit (Epicentre Biotechnologies, Madison, WI), which includes a DNase treatment step to eliminate genomic DNA as previously reported (18). Total RNA was also extracted from serial 1:10 dilutions of live *A. fumigatus* conidia (1 × 10^1^–1 × 10^9^) and DNase treated to form a standard curve. Lung *A. fumigatus* burden was analyzed with real-time PCR measurement of the *A. fumigatus* 18S rRNA (19) and quantified using a standard curve of *A. fumigatus* conidia as previously described (18). As a validation of the real-time PCR method, heat-killed *A. fumigatus* did not yield a signal by real-time PCR and were unable to grow on potato dextrose agar plates (18). In addition, no amplification controls (i.e., no reverse transcriptase included in the cDNA reaction) yielded a signal of <0.001% by real-time PCR, indicating that the DNase treatment step efficiently eliminated contaminating *A. fumigatus* DNA [as DNA is not predicative of organism viability (20)].

### 12-HETE analysis

Bronchoalveolar lavage was performed as previously described (21). 12-HETE levels were quantified using 12(S)-HETE ELISA Kit (catalog no. ADI-900-050) per the manufacturer’s instructions (Enzo Life Sciences, Farmingdale, NY).

### Inflammatory cytokine analysis

Mice were anesthetized with i.p. ketamine/xylazine and sacrificed by exsanguination at various time points postinfection. Both lungs were collected and minced in IMDM media (Sigma-Aldrich, St. Louis, MO) supplemented with 1% penicillin-streptomycin-glutamine (Mediatech, Herndon, VA), 10% heat-inactivated FBS (Invitrogen, Carlsbad, CA), and 0.4 mg/ml polymyxin B (Thermo Fisher Scientific), followed by incubation for 60 min with tissue culture-grade type IV collagenase (1 mg/ml; MilliporeSigma, St. Louis, MO) in a 37°C orbital shaker at 100 Rpm. The cell suspension was filtered through sterile 70- and 40-μm nylon filters and RBCs lysed with ammonium-chloride-potassium (ACK) buffer (Lonza, Walkersville, MD) to create lung cell digest preparations. For lung cell cultures, cells were enumerated on a hemacytometer and plated at 1 × 10^6^ cells in a volume of 0.2 ml. Supernatants were collected after 24 h, clarified by centrifugation and stored at −80°C. Supernatants were analyzed for protein levels of 32 cytokines and chemokines using a MILLIPLEX Multiplex Suspension Cytokine Array (MilliporeSigma) according to the manufacturer’s instructions. The data were analyzed using Bio-Plex Manager Software (Bio-Rad Laboratories, Hercules, CA). IL-23 and IL-22 levels were quantified by ELISA (R&D Systems). Serum was isolated using BD Microtainer Tubes (365967) according to the manufacturer’s instructions. G-CSF levels were quantified by MILLIPLEX assay or ELISA (R&D Systems, Minneapolis, MN).

### Flow cytometry

Lung cells were isolated previously as described above. Cells were washed, and Fc receptors were blocked with Mouse BD Fc Block (BD Biosciences, San Diego, CA) at 4°C for 20 min. Thereafter, cells were stained with a single-color LIVE/DEAD Fixable Dead Cell Stain (Invitrogen, Carlsbad, CA), followed by labeling with specific immune cell surface markers. The following staining parameters were employed: eosinophils as CD11b^+^ Siglec F^+^ Ly-6G^−^, neutrophils as CD11b^+^ Ly-6G^+^, inflammatory monocytes as CD11b^+^ Ly-6C^+^ CCR2^+^, invariant NKT (iNKT) cells as PBS57-labeled CD1d tetramer^+^, γδ T cells as γδ TCR^+^ CD3^+^ (all Abs purchased from BioLegend, eBioscience, and BD Biosciences; CD1d tetramer from National Institutes of Health Tetramer Core Facility, Emory University, Atlanta, GA). Bone marrow cells were isolated from one femur and tibia of naive or infected mice (22). Briefly, bones were harvested, cleaned of tissue, then centrifuged at 10,000 × *g* for 15 s to pellet bone marrow cells. Cell pellets were treated with ACK buffer to lyse RBCs and filtered through 40-μm nylon filters. Spleens were harvested, pushed through a 70-μm filter with a 3-ml syringe plunger, treated with ACK buffer, and subsequently filtered through a 40-μm filter. Blood was drawn from the inferior vena cava into blood collection tubes containing EDTA and lysed with ACK buffer. Analysis of PMN progenitor populations was performed as described (23). Briefly, dead cells and cells expressing CD3, CD4, CD8, B220, or Ter119 were excluded as well as side scatter-high, forward scatter-intermediate, and c-Kit^hi^CD34^low^ populations. Neutrophil precursors were then analyzed based on expression of c-Kit and Ly-6G.

### Lung injury assessment

A bronchoalveolar lavage was performed as previous described (21). Total protein levels were quantified using a Pierce BCA Protein Assay Kit. Lactate dehydrogenase levels were quantified using CytoTox 96 Non-Radioactive Cytotoxicity Assay (Promega, Madison, WI) per manufacturer’s instructions.

### Assessment of in vitro *A. fumigatus* killing

Neutrophils were isolated from the peritoneal cavity via thioglycolate elicitation as we have previously described (15). For *A. fumigatus* killing in vitro, neutrophils (1 × 10^5^) were cocultured at a 1:1 E:T ratio with live *A. fumigatus* swollen conidia for 24 h, followed by RNA isolation with the MasterPure Yeast RNA Purification Kit and real-time PCR assessment as described above. Controls included *A. fumigatus* swollen conidia cultured in the absence of neutrophils for 24 h. Alveolar macrophages were isolated by bronchoalveolar lavage as previously described (21) and cultured with resting conidia as above with neutrophils and analyzed similarly.

### Histology

The left lungs were collected and fixed in 4% formalin. The fixed lungs were paraffin embedded and then processed and stained by GNO Histology Consultants (New Orleans, LA). Imaging was performed using a Swift Optical Instruments M10T-P Trinocular LED Microscope equipped with a Motic Moticam 5+ 5 megapixel digital camera. The total number of organisms and number of germinated organisms were counted from two randomly selected fields per animal to compute percent germination.

### Statistics

Data were analyzed using GraphPad Prism Version 5.0 statistical software. Comparisons between groups when data were normally distributed were made with the Student *t* test. Significance was accepted at a *p* value < 0.05.

## Results

### 12/15-LOX deficiency results in profound mortality during *A. fumigatus* lung infection

**FIGURE 1.**
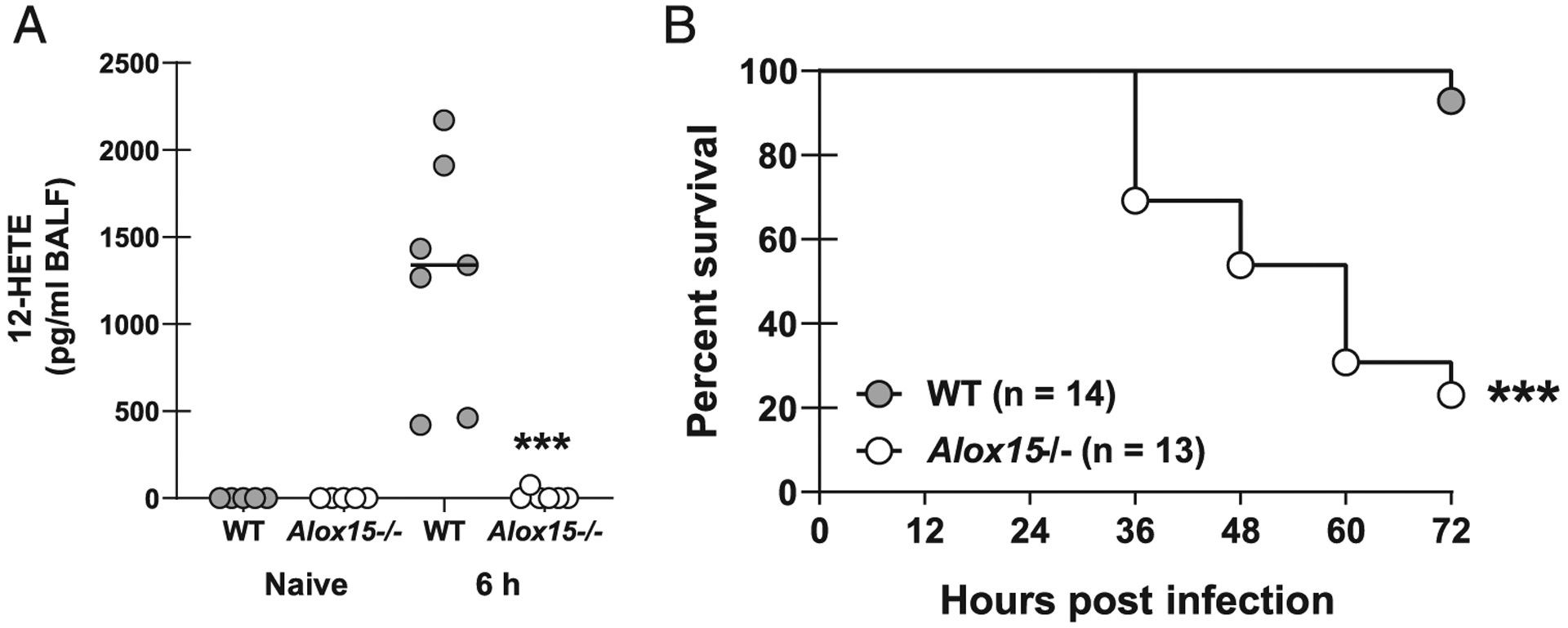
12/15-LOX deficiency results in profound mortality during *A. fumigatus* lung infection. (**A**) C57BL/6 WT and 12/15-LOX-deficient (*Alox15*^−/−^) mice were challenged intratracheally with 7 × 10^7^ *A. fumigatus* conidia. Levels of 12-HETE in bronchoalveolar lavage fluid (BALF) were quantified 6 h after challenge. Naive WT and *Alox15*^−/−^ mice served as controls. The figure illustrates cumulative data from two independent studies (*n* = 2–3 mice per group per study). (**B**) Mice were challenged as in (A) and monitored every 12 h for survival. The figure illustrates cumulative data from three independent studies (*n* = 4–5 mice per group per study). ***p < 0.001 (graph created by Kaplan-Meier estimator and the groups analyzed by the Mantel-Cox log-rank test and Gehan-Breslow-Wilcoxon test).

We have recently reported that in vivo pharmacologic inhibition of COX-2 results in impaired elimination of *A. fumigatus* from the lung (9). This correlated with a significant reduction in the production of multiple proinflammatory mediators previously reported to be required for fungal clearance (9). Another recent study reported that mice deficient in 5-LOX, which functions as a COX-2-independent arachidonic acid metabolism pathway leading to the generation of leukotrienes, exhibited decreased survival in response to a highly virulent *A. fumigatus* strain, but was dispensable for protection when challenged with a less-virulent strain (10). 12/15-LOX is an additional COX-2 independent arachidonic acid metabolism pathway (docosahexaenoic acid and linoleic acid may also serve as 12/15-LOX substrates) that generates lipoxins, resolvins, and protectins (11). Initial studies show that 12-HETE, a major product of 12/15-LOX activity, is rapidly induced in the lung after *A. fumigatus* exposure in a 12/15-LOX-dependent manner (Fig. 1A). Lung exposure to *A. fumigatus* resulted in rapid mortality in 12/15-LOX-deficient mice (*Alox15*^−/−^), with nearly a third of the mice succumbing to infection within 36 h and only 20% of mice surviving by 72 h (Fig. 1B). The survival defect was apparent in both male and female mice (data not shown). Thus, 12/15-LOX is essential for survival after acute challenge with *A. fumigatus*.

### The absence of 12/15-LOX results in impaired fungal clearance and increased lung damage during *A. fumigatus* lung infection

**FIGURE 2.**
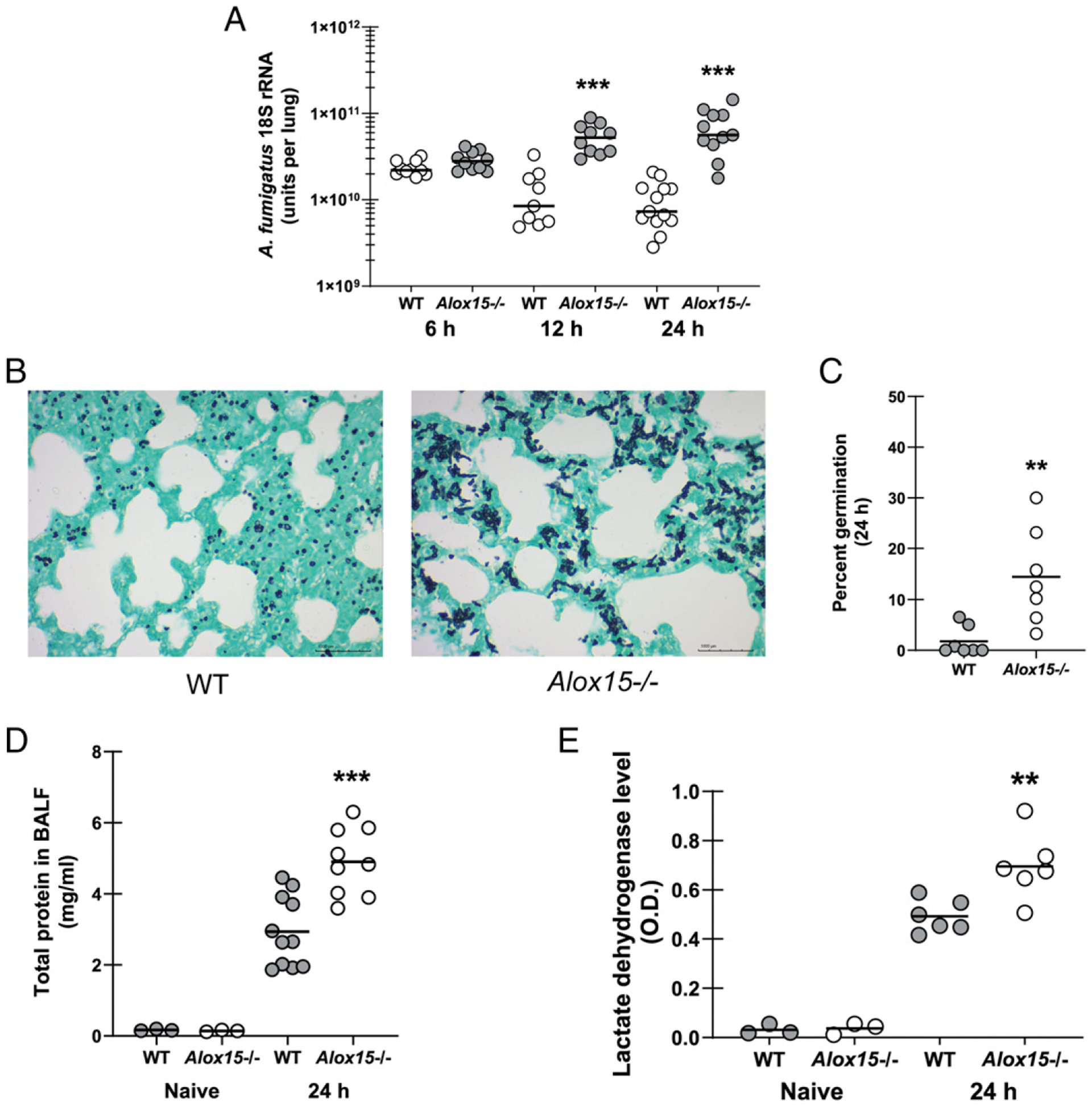
The absence of 12/15-LOX results in impaired fungal clearance and increased lung damage during *A. fumigatus* lung infection. C57BL/6 WT and 12/15-LOX-deficient (*Alox15*^−/−^) mice were challenged intratracheally with 7 × 10^7^ *A. fumigatus* conidia. (**A**) Lung fungal burden at 6, 12, and 24 h postchallenge was assessed by real-time PCR analysis of *A. fumigatus* 18S rRNA levels. The figure illustrates cumulative data from two to three independent studies (*n* = 4–6 mice per group per study). Each circle represents an individual mouse. Line within a given group represents the mean. (**B**) Representative Grocott-Gomori’s methenamine silver (GMS)-stained lung sections from WT mice (left) and *Alox15*^−/−^ mice (right). Original magnification ×40. Scale bar, 1000 μm. (**C**) Percent germination was calculated by enumerating the total number of organisms and the number of germinated organisms from two randomly selected fields per animal (*n* = 2–3 mice per group). (**D** and **E**) Bronchoalveolar lavage was performed with 1 ml of PBS at 24 h postchallenge. The clarified BALF was analyzed for (D) total protein and (E) lactate dehydrogenase levels. The figure illustrates cumulative data from two to three independent studies (*n* = 3–4 mice per group per study). Controls include naive mice (*n* = 1–2 per group per study). Each circle represents an individual mouse. Line within a given group represents the mean. **p < 0.01, ***p < 0.001 (unpaired two-tailed Student *t* test).

Based on the observation that 12/15-LOX-deficient mice demonstrated rapid and significant mortality after lung challenge with *A. fumigatus*, we hypothesized that these mice had difficulty clearing *A. fumigatus* from the lungs. Although lung fungal burden was similar 6 h after *A. fumigatus* challenge, *Alox15*^−*/*−^ mice demonstrated a 4-fold and 7-fold higher fungal burden at 12 and 24 h after exposure, respectively (Fig. 2A). Grocott-Gomori’s methenamine silver staining of lung tissues also indicated higher numbers of *A. fumigatus* organisms in *Alox15*^−*/*−^ mice (Fig. 2B) as well as more germinating organisms (Fig. 2C). As germinating organisms can augment lung injury, we further show that total protein (Fig. 2D), a measure of lung leakage, and lactate dehydrogenase (Fig. 2E), a measure of lung cell damage/death, levels were elevated in lavage fluid from 12/15-LOX-deficient mice 24 h postexposure. These data suggest that mortality associated with 12/15-LOX deficiency is a result of lung damage induced by elevated fungal burden.

### 12/15-LOX is required for early inflammatory responsiveness during *A. fumigatus* lung infection

**FIGURE 3.**
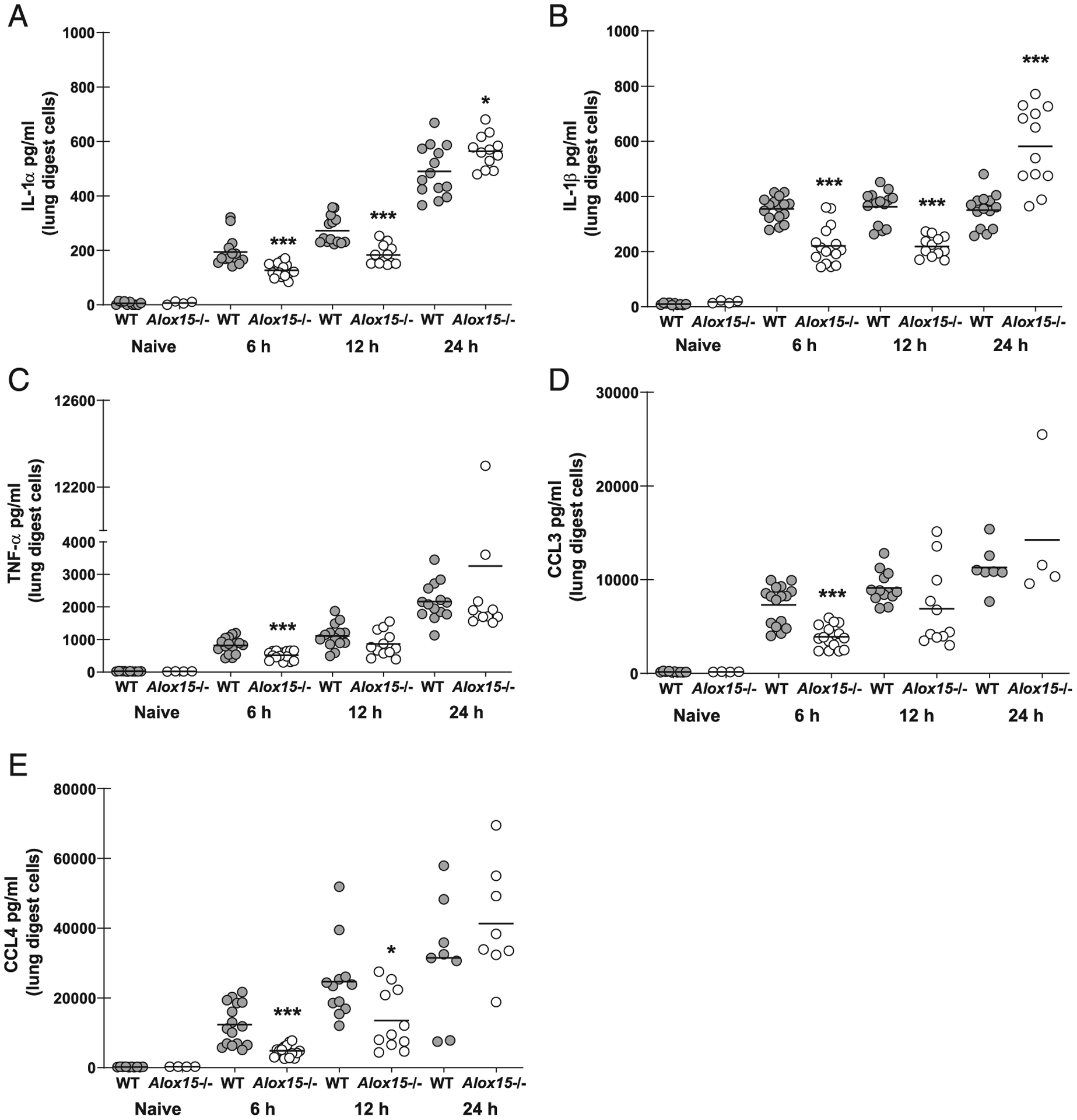
12/15-LOX is required for early inflammatory responsiveness during *A. fumigatus* lung infection. C57BL/6 WT and 12/15-LOX-deficient (*Alox15*^−/−^) mice were challenged intratracheally with 7 × 10^7^ *A. fumigatus* conidia. At 6, 12, and 24 h postchallenge, lungs were collected, enzymatically digested, and cultured in duplicate for 24 h. (**A**) IL-1α, (**B**) IL-1β, (**C**) TNF-α, (**D**) CCL3, and (**E**) CCL4 levels were quantified in coculture supernatants by Luminex-based MILLIPLEX assessment. The figure illustrates cumulative data from two independent studies (*n* = 3–4 mice per group per time point per study). Controls include naive mice (*n* = 1–2 per group per study). Each circle represents an individual sample. Line within a given group represents the mean. *p < 0.05, ***p < 0.001 (unpaired two-tailed Student *t* test).

*A. fumigatus* exposure results in the induction of multiple proinflammatory responses that are required for pathogen elimination (24–26). However, in some instances, these proinflammatory responses may complicate fungal clearance (27, 28). As demonstrated earlier, fungal clearance was impaired in *Alox15*^−/−^ mice as early as 12 h after *A. fumigatus* exposure, suggesting that 12/15-LOX was critical for an early proinflammatory response that promoted inflammatory cell recruitment. To this end, results show that by 6 h after *A. fumigatus* challenge, *Alox15*^−/−^ mice had significant reductions in IL-1α (Fig. 3A), IL-1β (Fig. 3B), TNF-α (Fig. 3C), CCL3 (Fig. 3D), and CCL4 (Fig. 3E). IL-1α, IL-1β, and CCL4 levels in *Alox15*^−/−^ mice remained lower 12 h after *A. fumigatus* exposure. By 24 h after challenge, however, multiple mediators were increased in *Alox15*^−/−^ mice, likely as a result of the heightened fungal burden (CCL3 and CCL4 in particular, levels of which were often beyond the limit of detection in our assay). CXCL1 was not increased over naive levels, whereas CXCL2 was not different between wild-type (WT) and *Alox15*^−/−^ (data not shown). Taken together, these data suggest that 12/15-LOX is required for the optimal early induction of proinflammatory cytokines and chemokines that support neutrophil recruitment to the lung during *A. fumigatus* infection.

### 12/15-LOX is required for inflammatory cell recruitment during *A. fumigatus* lung infection

**FIGURE 4.**
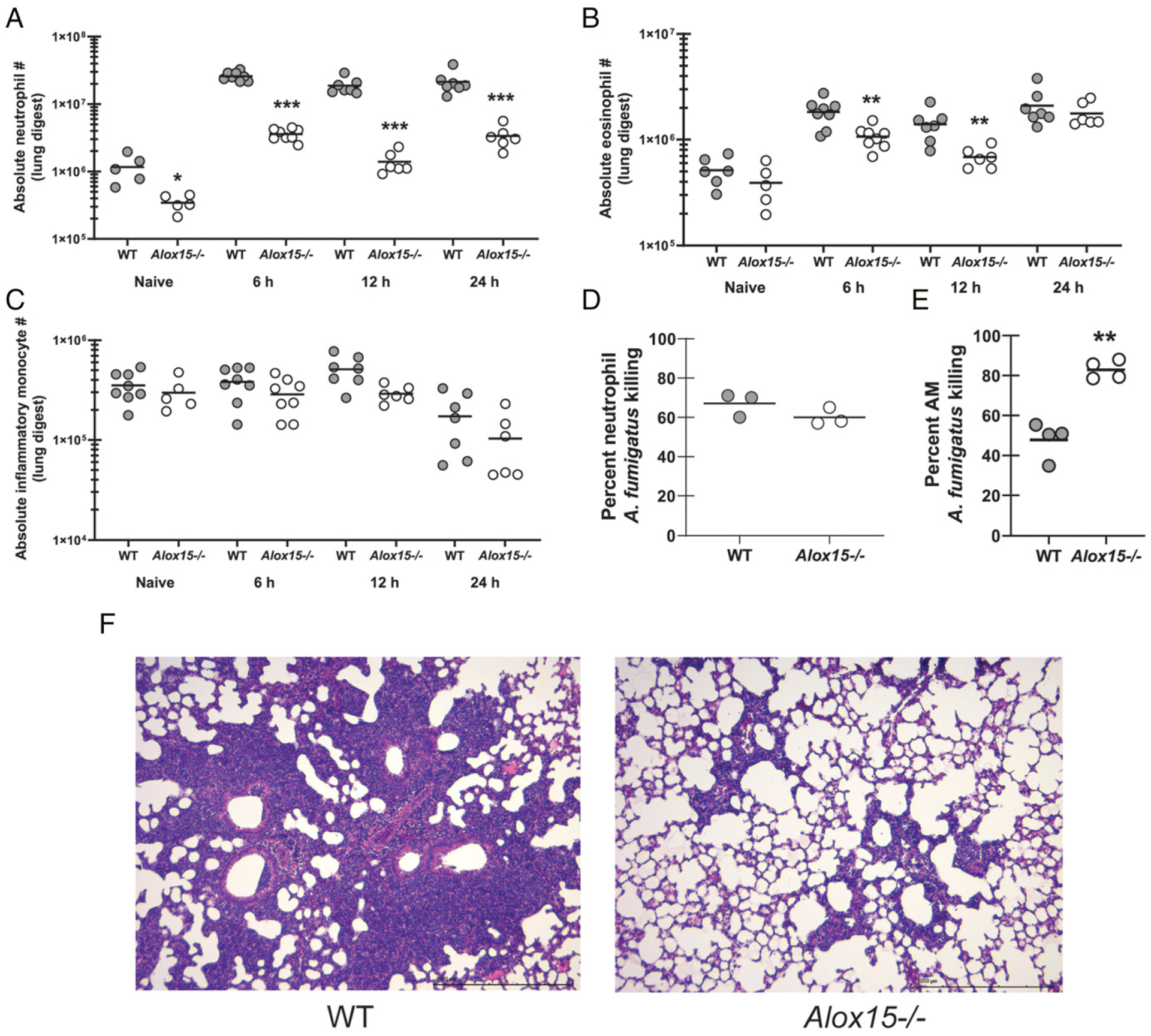
12/15-LOX is required for inflammatory cell recruitment during *A. fumigatus* lung infection. C57BL/6 WT and 12/15-LOX-deficient (*Alox15*^−/−^) mice were challenged intratracheally with 7 × 10^7^ *A. fumigatus* conidia. At 6, 12, and 24 h postchallenge, lungs were collected, enzymatically digested, and (**A**) neutrophils (CD11b^+^ Ly-6G^+^), (**B**) eosinophils (CD11b^+^ Siglec F^+^), and (**C**) inflammatory monocytes (CD11 b^+^ Ly-6C^+^ CCR2^+^) were quantified by flow cytometry. The figure represents cumulative data from two independent studies (*n* = 3–4 mice per group per time point per study). Controls include naive mice (*n* = 2–3 per group per study). Each circle represents an individual mouse. Line within a given group represents the mean. (**D**) Thioglycolate-elicited neutrophils or (**E**) alveolar macrophages from WT and *Alox15*^−/−^-deficient were plated 1:1 with *A. fumigatus* conidia. Total contents of each well were collected, and fungal killing was assessed by real-time PCR analysis of *A. fumigatus* 18S rRNA levels. Data are expressed as percentage of fungi killed. The figure illustrates cumulative data from two to three independent studies (*n* = 1–2 mice per group per study). Each circle represents an individual mouse. Line within a given group represents the mean. (**F**) Representative H&E-stained lung sections from WT mice (left) and *Alox15*^−/−^ mice (right). Original magnification ×10. Scale bar, 1000 μm. *p < 0.05, **p < 0.01, ***p < 0.001 (unpaired two-tailed Student *t* test).

Neutrophils are a vital component of the innate immune response to IA. Neutropenia, defective recruitment to infected tissues or the inability of neutrophils to mediate antifungal activities are well-recognized contributors to the development of IA (29). We rationalized that the development of IA in 12/15-LOX-deficient mice was a result of impaired neutrophil recruitment or activity. Indeed, as early as 6 h after *A. fumigatus* challenge, a time point in which fungal burden was similar between WT and *Alox15*^−/−^ mice (Fig. 2), we observed 5-fold lower numbers of neutrophils in the lungs of *Alox15*^−/−^ mice (Fig. 4A). This defect in lung neutrophil levels was maintained through 12 and 24 h after *A. fumigatus* challenge (Fig. 4A). Studies by us and others have previously identified antifungal roles for eosinophils (30) and inflammatory monocytes (31) in innate immune clearance of *A. fumigatus*. Eosinophil recruitment was dependent on 12/15-LOX at 6 and 12 h after *A. fumigatus* challenge but was restored to WT levels by 24 h (Fig. 4B). In contrast, we observed no dependency on 12/15-LOX for inflammatory monocyte recruitment at any time point (Fig. 4C). Of note, lung neutrophils, but not eosinophils and inflammatory monocytes, were significantly lower in naive *Alox15*^−/−^ mice (Fig. 4A–C). Despite lower numbers in the lungs, *Alox15*^−/−^ neutrophils did not demonstrate an impairment in in vitro antifungal activity (Fig. 4D). Unexpectedly, however, alveolar macrophages from naive *Alox15*^−/−^ mice had enhanced antifungal activity against *A. fumigatus* (Fig. 4E), suggesting that 12/15-LOX plays a restrictive role in macrophage antifungal activity. H&E staining confirmed the profound reduction in inflammatory cell infiltration in the lungs of 12/15-LOX-deficient mice (Fig. 4F). Collectively, 12/15-LOX is required for optimal neutrophil numbers in the lung; however, it is dispensable for neutrophil antifungal activity. Moreover, increased antifungal activity by alveolar macrophages is not sufficient to compensate for the reduced neutrophil numbers in the lung in *Alox15*^−/−^ mice.

### 12/15-LOX is required for neutrophil granulopoiesis during *A. fumigatus* lung infection

**FIGURE 5.**
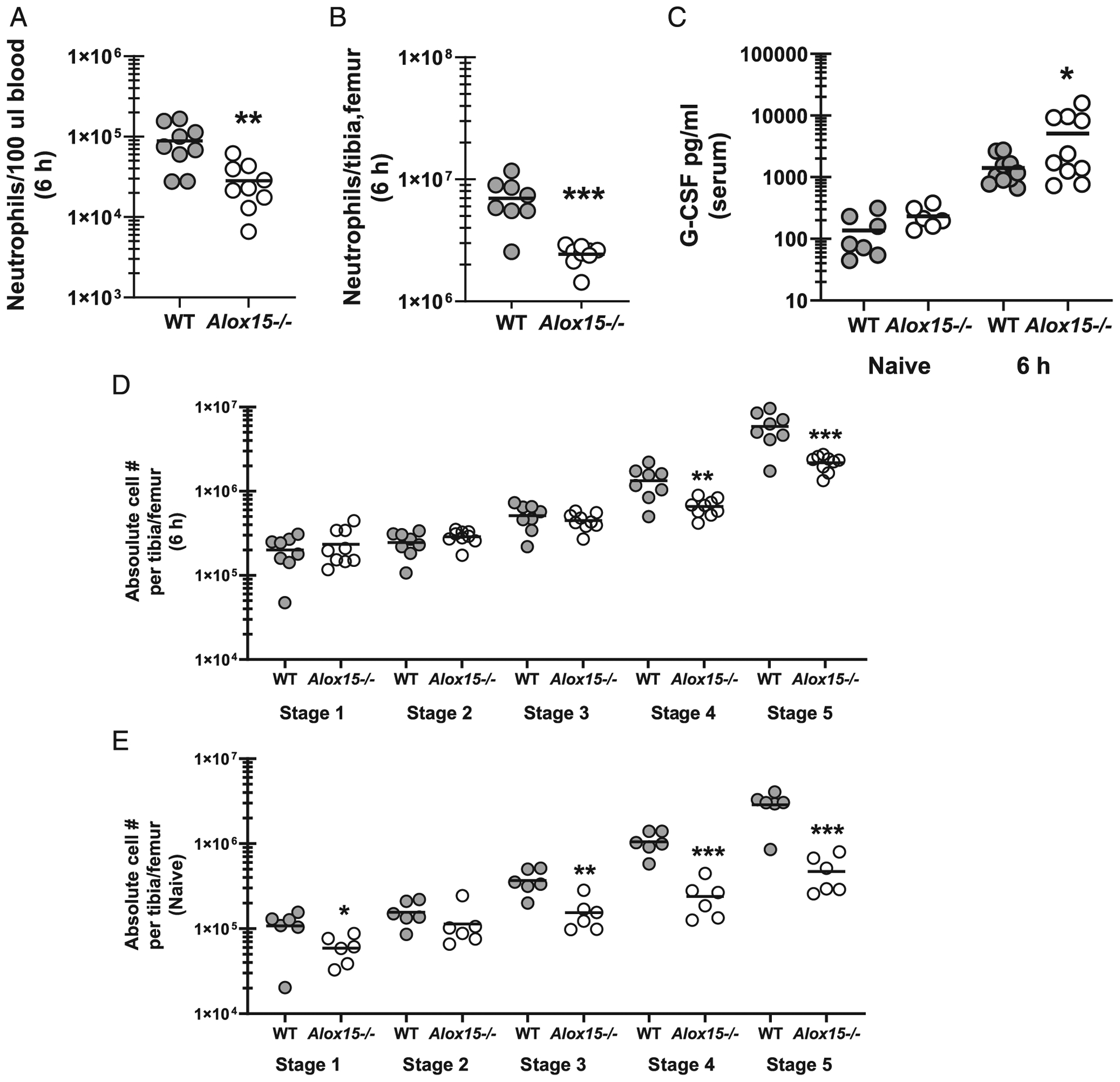
12/15-LOX is required for neutrophil granulopoiesis during *A. fumigatus* lung infection. C57BL/6 WT and 12/15-LOX-deficient (*Alox15*^−/−^) mice were challenged intratracheally with 7 × 10^7^ *A. fumigatus* conidia. (**A**) Neutrophils (CD11b^+^ Ly-6G^+^) were quantified in blood and (**B**) bone marrow 6 h after challenge by flow cytometry. The figure represents cumulative data from two independent studies (*n* = 4–5 mice per group per time point per study). Each circle represents an individual mouse. Line within a given group represents the mean. (**C**) G-CSF levels were quantified in serum by ELISA. The figure represents cumulative data from two independent studies (*n* = 4–5 mice per group per time point per study). Controls include naive mice (*n* = 2–3 per group per study). Each circle represents an individual mouse. Line within a given group represents the mean. (**D** and **E**) Neutrophil precursors (five stages differentiated by c-Kit and Ly-6G^+^ expression) were quantified in bone marrow (D) 6 h after challenge or (E) in naive mice by flow cytometry. The figure represents cumulative data from two independent studies (*n* = 4–5 mice per group per time point per study). Each circle represents an individual mouse. Line within a given group represents the mean. *p < 0.05, **p < 0.01, ***p < 0.001 (unpaired two-tailed Student *t* test).

We speculated that lower induction of proinflammatory cytokines and chemokines (Fig. 3) was likely responsible for the observed reduction in neutrophils in the lungs of *Alox15*^−/−^ mice. However, we also observed reduced neutrophil numbers in the blood (Fig. 5A) and bone marrow (Fig. 5B) from *Alox15*^−/−^ mice 6 h after *A. fumigatus* challenge. This observation suggested the possibility that neutrophil survival, which is controlled by G-CSF, may be impaired in *Alox15*^−/−^ mice. However, G-CSF levels in serum from naive *Alox15*^−/−^ mice was comparable to naive WT mice and even slightly increased by 6 h after *A. fumigatus* challenge (Fig. 5C). To further understand lower neutrophil levels in multiple compartments in *Alox15*^−/−^ mice, we examined granulocyte differentiation via c-Kit and Ly-6G expression in the bone marrow. During the five-stage process of granulocyte differentiation and acquisition of granule contents, granulocytes acquire Ly-6G expression and gradually lose the expression of c-Kit (23). We found that 6 h after *A. fumigatus* challenge, *Alox15*^−/−^ mice had similar numbers of myeloblasts, promyelocytes, and myelocytes (stages 1, 2, and 3) compared with WT mice (Fig. 5D). In contrast, metamyelocytes (stage 4) and mature neutrophils (stage 5) were significantly lower in *Alox15*^−/−^ mice compared WT mice (Fig. 5D). Defective neutrophil maturation in the bone marrow was intrinsic to *Alox15*^−/−^ mice, as naive mice also demonstrated lower numbers of myelocytes, metamyelocytes, and mature neutrophils (Fig. 5E). Taken together, these results suggest that impaired neutrophil levels in the lungs of *Alox15*^−/−^ mice are a result of 12/15-LOX-dependent neutrophil granulopoiesis in the bone marrow rather than a defect in recruitment or survival.

### Type 17 responses during *A. fumigatus* lung infection are dependent on 12/15 LOX

**FIGURE 6.**
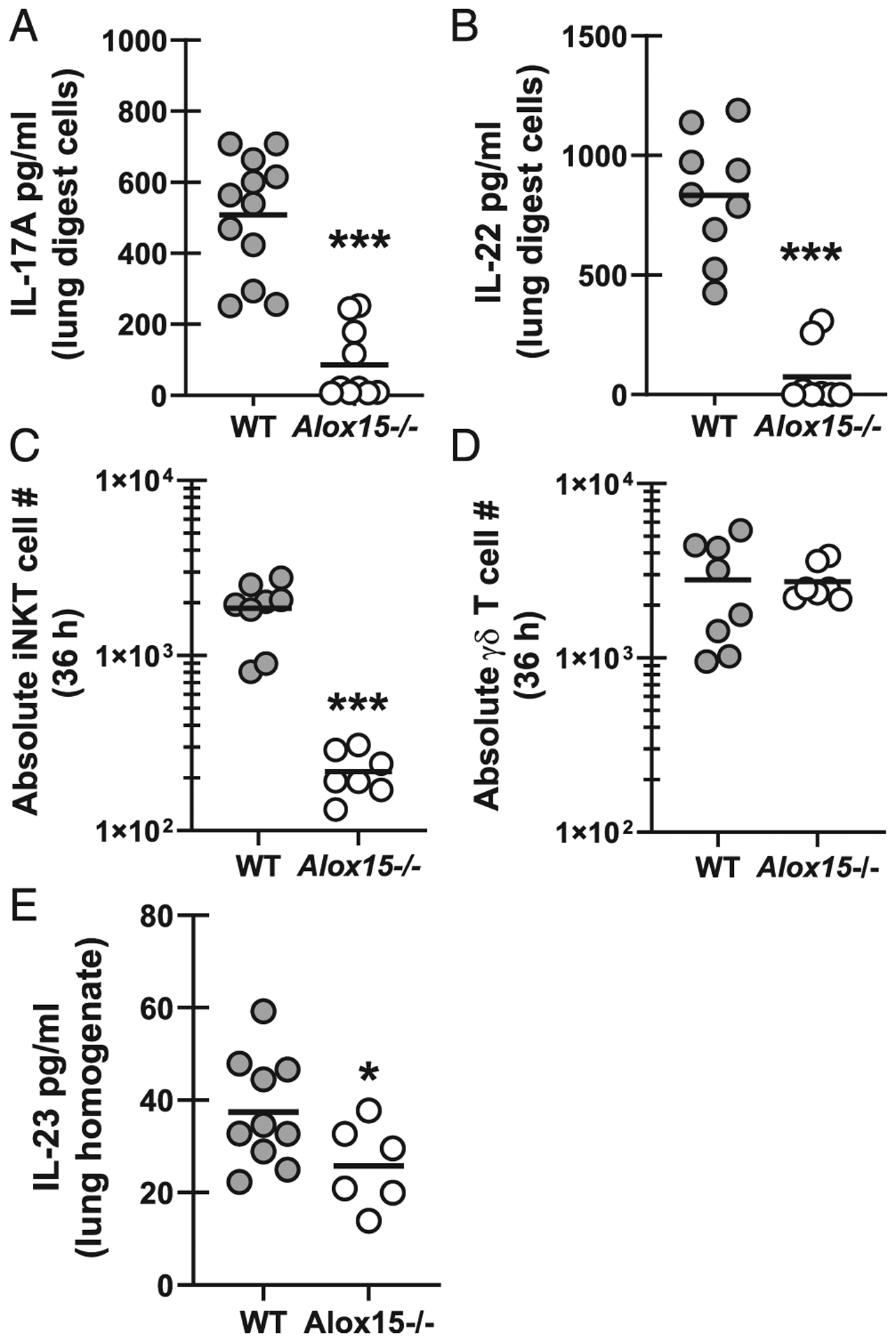
Type 17 responses during *A. fumigatus* lung infection are dependent on 12/15 LOX. C57BL/6 WT and 12/15-LOX-deficient (*Alox15*^−/−^) mice were challenged intratracheally with 4 × 10^7^ *A. fumigatus* conidia. At 40 h postchallenge, the right lungs were collected, enzymatically digested, and cultured in duplicate for 24 h. (**A**) IL-17A and (**B**) IL-22 levels were quantified in coculture supernatants by Luminex-based MILLIPLEX assessment or ELISA. The figure illustrates cumulative data from three independent studies (*n* = 2–3 mice per group per study). Each circle represents an individual sample. Line within a given group represents the mean. (**C** and **D**) At 36 h postchallenge, lungs were collected, enzymatically digested, and (C) iNKT cells (PBS57-labeled CD1d tetramer^+^), and (D) γδ T cells (γδ TCR^+^ and CD3^+^) were quantified by flow cytometry. The figure represents cumulative data from three independent studies (*n* = 2–3 mice per group per study). Each circle represents an individual mouse. Line within a given group represents the mean. (**E**) Mice were challenged as described. At 40 h postchallenge, the left lungs were collected and homogenized in 1 ml of PBS supplemented with pro-tease inhibitors. IL-23 was quantified in clarified homogenate supernatants by ELISA. The figure illustrates cumulative data from two to three independent studies (*n* = 3–4 mice per group per study). Each circle represents an individual mouse. Line within a given group represents the mean. *p < 0.05, ***p < 0.001 (unpaired two-tailed Student *t* test).

We have previously reported that IL-17A and IL-22 are critical for lung defense during IA (15, 16). Recently, we have reported that iNKT cells and γδ T cells are the primary sources of IL-22 during IA (32) and hypothesize that these cell types may also serve as lung cell sources of IL-17A. We have shown that IL-22 production peaks in the lung 48 h after *A. fumigatus* challenge (32). To determine the impact of 12/15-LOX on type 17 (IL-17A and IL-22) responses during IA, we challenged mice with a lower inoculum, thus allowing for detection of IL-17A and IL-22 at time points between 36 and 48 h. Results demonstrated that IL-17A (Fig. 6A) and IL-22 (Fig. 6B) were highly dependent on 12/15-LOX. To further investigate mechanisms associated with lower levels of IL-17A and IL-22, we examined the presence of innate-like lymphocytes in the lung, which demonstrated reduced numbers of iNKT cells (Fig. 6C), but not γδ T cells (Fig. 6D). Reduced iNKT cell numbers did not correlate with reductions in CCL17 (data not shown), a chemokine implicated in iNKT cell extravasation from the vasculature to the lung (33). Finally, we also observed lower levels of IL-23, which we have previously reported as a central upstream mediator of type 17 responses (15, 16), in the lungs of *Alox15*^−/−^ mice after *A. fumigatus* challenge (Fig. 6E). Thus, 12/15-LOX is critical for induction of type 17 responses during IA, likely through the induction of IL-1β and IL-23.

## Discussion

Detailed understanding of how the innate immune system deploys and organizes the response to *A. fumigatus* in normal hosts is fundamental to developing new immune-based therapies against IA. Of particular interest regarding the lung, resolution of the inflammatory response is nearly as important as induction of inflammation in terms of a successful response to infection. Arachidonic acid is a polyunsaturated fatty acid released from the cell membrane by phospholipases in response to various inflammatory conditions (34). Cyclooxygenases, cytochrome P450 mono-oxygenases, and LOXs are the enzymes involved in the oxidative metabolism of arachidonic acid, the latter of which leads to the production of leukotrienes, hydroperoxyeicosatetraenoic acids, HETEs, and hydroxyoctadecadienoic acids (35). The major LOXs in humans include 5-LOX, 12-LOX (platelet and leukocyte forms), and 15-LOX (reticulocyte and leukocyte forms), although leukocyte 12-LOX and reticulocyte 15-LOX form similar products and, thus, are usually referred to as 12/15-LOX (35). Mice express 5-LOX and 12/15-LOX (leukocyte 12-LOX), which is encoded by the *Alox15* gene and is considered orthologous to human 12/15-LOX. Select lipid species, such as lipoxin A4 produced by 12/15-LOX, have noted proresolution functions and are thought to be produced later in a given response in an effort to temper inflammation and damage (36). Recent reports have demonstrated that diverse arachidonic acid metabolism pathways, specifically the induction of COX-2 and 5-LOX, are critical components of the acute inflammatory response to *A. fumigatus* in the lung (9, 10). In this study, we interrogated the role of the 12/15-LOX pathway in regulating the immune response to a highly inflammatory opportunistic mold.

Our initial findings revealed that *Alox15*^−/−^ mice were profoundly susceptible to mortality after lung exposure to *A. fumigatus*, demonstrating ~20% survival within 72 h postchallenge. This level of susceptibility rivals what we have previously reported for mice deficient in Dectin-1, a β-glucan-specific pattern recognition receptor required for lung defense against *A. fumigatus* (15), and was more severe than that recently reported for 5-LOX-deficient mice (10). We primarily attributed mortality in *Alox15*^−/−^ mice to impaired lung fungal clearance, as fungal burden was >7-fold higher in deficient mice by 24 h. Grocott-Gomori’s methenamine silver staining of lung tissue suggested increased fungal germination in the alveolar space, and along with enhanced fungal burden, these observations corresponded with markers of lung injury. Immunologic assessments correlating with defective fungal clearance in *Alox15*^−/−^ mice included defective IL-1α and IL-1β production. Renewed interest in the IL-1 family of cytokines has uncovered novel roles for IL-1α and IL-1β in lung defense during acute *A. fumigatus* exposure (24, 25). Although we have previously reported that IL-1α is dependent on IL-22 after acute *A. fumigatus* exposure (16), we have also recently reported that signaling through the IL-1R is required for optimal IL-22 production after *A. fumigatus* exposure (9). IL-1α is thought to mediate protection via CXCL1-dependent neutrophil recruitment (24). Although CXCL1 levels were not induced over the levels observed in naive mice, we instead observed defective production of the neutrophil attracting chemokines CCL3 and CCL4. Mice deficient in CCR1, the receptor for CCL3 and CCL4, display defective neutrophil recruitment to the lung and decreased survival after *A. fumigatus* challenge, highlighting the importance of CCL3 and CCL4 signaling during IA (37). Moreover, our previous work has demonstrated reduced neutrophil recruitment in the presence of impaired TNF-α, CCL3, and CCL4 production in Dectin-1-deficient mice during IA (15). Furthermore, we have shown that TNF-α, CCL3, and CCL4 production are impaired in IL-22-deficient mice, which also demonstrate impaired neutrophil recruitment and increased susceptibility to IA (16). Collectively, defective induction of the IL-1α/IL-1β, TNF-α, and CCL3/CCL4 axes in the initial hours following *A. fumigatus* are likely major contributors to the overall susceptibility of *Alox15*^−/−^ mice to IA. Ongoing studies in our laboratory are investigating the relationship between pattern recognition receptor recognition of *A. fumigatus* and activation of 12/15-LOX and related pathways to promote the inflammatory responses we report in this study.

Despite the fact that the IL-1α/IL-1β, TNF-α, and CCL3/CCL4 axes play a role in neutrophil recruitment to the lungs during *A. fumigatus* infection and the fact that these axes are 12/15-LOX dependent, our data suggest a more compelling role for 12/15-LOX in neutrophil responses during *A. fumigatus* infection. Previous reports have shown that naive, 6–8-wk-old *Alox15*^−/−^ mice have no major differences in the levels of immune cell populations in bone marrow and blood (38, 39). In contrast, other studies have reported that naive *Alox15*^−/−^ mice at ~15 wk of age have changes in splenic immune cell populations, specifically decreased lymphocytes and monocytes, increased basophils, but no differences in neutrophils (40). However, a follow-up study by the same group reported that naive *Alox15*^−/−^ mice had evidence for paradoxically enhanced granulopoiesis (increased Ly-6G^+^ cells and increased GMPs in the bone marrow) (41). In contrast, similar to our 6 h infection data, we observed that naive *Alox15*^−/−^ mice (at 6–8 wk old, which were employed for all studies in the current body of work) demonstrated lower levels of myelocytes (stage 3), metamyelocytes (stage 4), and mature neutrophils (stage 5) in the bone marrow. Other models of lung infection with *Mycobacterium tuberculosis* (42) and *Streptococcus pneumoniae* (14) have reported reductions in lung neutrophil numbers in *Alox15*^−/−^ mice; however, these reports hypothesized that lower levels of neutrophils were a result of impaired recruitment. Although the *M. tuberculosis* study (42) performed bone marrow chimera studies and demonstrated lower neutrophils in the lungs of *M. tuberculosis*-infected mice receiving *Alox15*^−/−^ bone marrow, their interpretation was that *Alox15* expression in a hematopoietic cell was required for neutrophil recruitment to the lungs during infection. With our study investigating neutrophils levels in the bone marrow of both naive and infected mice as well as the numbers of specific neutrophil precursors, it is, to our knowledge, the first to suggest that lower neutrophil numbers during lung infection may be the result of failed neutrophil granulopoiesis in the bone marrow of *Alox15*^−/−^ mice rather than failed recruitment or survival.

The cytokines IL-17A and IL-22 promote microbial clearance via the induction of chemokines that support neutrophil recruitment as well as the elicitation of antimicrobial factors from the epithelium (43). We have shown in numerous reports the importance of IL-17A and IL-22 in lung defense against *A. fumigatus* (15, 16, 44). However, these studies were conducted using our standard inoculum for nonimmunosuppressed mice. At this concentration, *Alox15*^−/−^ mice did not survive to the time point in which we have reported IL-17A and IL-22 to be maximally expressed (i.e., 36–48 h) (32); thus, we were unable to thoroughly investigate the impact of the 12/15-LOX pathway on IL-17A/IL-22 responses. However, by lowering the inoculum, we were able to document that IL-17A and IL-22 production were 12/15-LOX dependent. Results explaining the defect in IL-17A and IL-22 production showed that IL-23, an upstream positive regulator we have shown to be required for IL-17A (44) and IL-22 (16) during IA, was partially 12/15-LOX dependent. We have recently reported that innate-like lymphocytes, specifically iNKT cells and γδ T cells, are the primary cell sources of IL-22 (and likely IL-17A) in the lung during IA (32). An additional mechanism of lower IL-17A and IL-22 has fewer iNKT cell, but not γδ T cell, numbers in the lungs of *Alox15*^−/−^ mice. Based on observing lower numbers of iNKT cells in the spleen of naive *Alox15*^−/−^ mice (data not shown) and intact levels of the iNKT chemokine CCL17 (data not shown) in the lung during IA, it is tempting to speculate whether iNKT cell development as opposed to 12/15-LOX-mediated recruitment is impaired in *Alox15*^−/−^ mice during infection. Our findings extend observations in the gut, showing lower IL-17A levels in *Alox15*^−/−^ mice during peritonitis (45), likely as a result of impaired macrophage-derived IL-23 production (46).

In many lung model systems, the initial inflammatory wave is characterized by COX-2-mediated PGE_2_ and 5-LOX-mediated leukotriene production in concert with neutrophil recruitment (47). As inflammation ensues, 12/15-LOX-mediated pathways are engaged to drive resolution of the inflammatory response via specialized proresolving mediators (lipoxins, resolvins, protectins, and maresins). Our data points to a requirement for 12/15-LOX activity in the induction of very early antifungal innate immune response following exposure to *A. fumigatus*, namely inflammatory cytokine and chemokine induction. Strikingly, however, our data uncover a new role for 12/15-LOX in driving neutrophil granulopoiesis in the bone marrow. As these responses occur within hours after fungal exposure, we conclude that 12/15-LOX has a conclusive proinflammatory role in the acute inflammatory response to *A. fumigatus*. To this end, our study provides new insight into signaling events catalyzing early innate immune activity during invasive lung fungal infections.

## Abbreviations used in this article:

ACK: ammonium-chloride-potassium
HETE: hydroxyeicosatetraenoic acid
IA: invasive aspergillosis
iNKT: invariant NKT
LOX: lipoxygenase
WT: wild-type
